# Free to be: Experiences of arts-based relational caring in a community living and thriving with dementia

**DOI:** 10.1177/14713012211027016

**Published:** 2021-06-24

**Authors:** Christine Jonas-Simpson, Gail Mitchell, Sherry Dupuis, Lesley Donovan, Pia Kontos

**Affiliations:** 7991 York University, Toronto, ON, Canada; 8430 University of Waterloo, Waterloo, ON, Canada; St. Michael’s Hospital, 508783 Unity Health Toronto, Toronto, ON, Canada; KITE-Toronto Rehabilitation Institute, University Health Network, Toronto, ON, Canada; Dalla Lana School of Public Health, University of Toronto, Toronto, ON, Canada

**Keywords:** persons living with dementia, dementia care, relational caring, arts-based curriculum, compassionate care, community care, creative expression, long-term care, thriving with dementia

## Abstract

**Aim:**

To present findings about experiences of relational caring at an arts-based academy for persons living with dementia.

**Background:**

There is a compelling call and need for connection and relationships in communities living with dementia. This study shares what is possible when a creative arts-based academy for persons living with dementia grounded in relational inquiry and caring focuses on relationships through the medium of the arts.

**Design:**

A qualitative phenomenological methodology (informed by van Manen) was used to answer the research question, “What is it like to experience relational caring at an arts-based academy for persons living with dementia?” We address two research objectives: (1) to explore how relationships are experienced when a relational caring philosophy underpins practice, including arts-based engagements; and (2) to understand the meaning of relationships that bring quality to day-to-day living.

**Methods:**

Twenty-five participants were recruited from the Academy and interviewed in one-to-one in-depth interviews or small groups. Participants included five persons living with dementia, eight family members, four staff, five artists, one personal support worker, and two volunteers. Participants were asked to describe their experiences of relational caring or relationships in the Academy space.

**Findings:**

Three thematic patterns emerged, which address the research objectives.

Relational caring is experienced when:

*freedom and fluid engagement inspire a connected spontaneous liveliness*;
*embracing difference invites discovery and generous inclusivity*; and
*mutual affection brings forth trust and genuine expression*.

**Conclusions:**

Findings contribute to the growing body of knowledge about both relational caring and arts-based practices that call forth a different ethic of care—one that is relational, inclusive, and intentional. Findings also shed light on what is possible when a relational caring philosophy underpins arts-based practices—everyone thrives.

Relational caring is a philosophy of care that is evolving in response to a predominant focus on person-centered care, which has the propensity to decontextualize and isolate the individual from his or her relational world (Dupuis, Gillies, et al., 2012; Kontos, Miller, & Kontos, 2017; Mitchell, Dupuis, et al., 2020; Mitchell, Jonas-Simpson, et al., 2020). Relational caring respects the importance of person-centeredness and embeds it deeply within relationships (Dupuis, Gray, et al., 2016, Dupuis et al., 2018). We agree with Bellass et al. (2019) that the “person-centered paradigm, while enacting a welcome shift in emphasis from the biomedical features of dementia to the person living with the condition, has arguably concretized an individual, rather than a relational focus” (p. 2802). Thus, there is an urgent need to build on efforts of others (see for example, Abma & Baur, 2014; Basting, 2018; Hamington, 2015; Jennings, 2009a, 2009b) to foster connection and opportunities for authentic relationships, especially among persons living with dementia who are often excluded in communities as active valued citizens because of stigma (Dupuis, Gillies, et al., 2012; Kontos, Miller, & Kontos, 2017; Mitchell et al., 2013).

Further, persons and families may live with dementia for many years. It is imperative that new initiatives focus on enhancing the quality of day-to-day living in ways that honour embodied selfhood (Kontos, 2012a, 2012b), lifelong learning, and relationships, where everyone thrives. Our research advances this agenda by providing insights about how people *experience* relational caring at an arts-based wellness academy for persons living with dementia. Our purpose here is to report findings from a phenomenological study that explored how individuals affiliated with an arts-based academy for persons living with dementia (hereafter referred to as the Academy) experienced relational caring or relationships.

## Background

### Relational approaches to caring

The call for a new ethic of care for older adults and especially persons living with dementia is pervasive in the literature (Abma & Baur, 2014; Armstrong & Lowndes, 2018; Beach & Inui, 2006; Dupuis, McAiney, et al., 2016; Jonas-Simpson et al., 2012; Kontos et al., 2016; Kontos, Miller, & Kontos, 2017; Kontos et al., 2020; Mitchell, Dupuis, et al., 2020, 2021; Nolan et al., 2002, 2004). Nolan et al. (2002, 2004) were among the first to emphasize the relational embeddedness of care and how person-centered approaches failed to recognize the interdependent and reciprocal nature of care in the dementia care context. Nolan et al. (2006) identified six essentials for relationship-centered care (RCC): security, belonging, continuity, purpose, achievement, and significance. These six senses should be experienced by all in care, including older adults, family members, and formal care providers for quality of life and well-being. Similarly, Beach and Inui (2006) identified the importance of RCC as a framework for conceptualizing all health care where the quality of genuine reciprocal relationships are valued and are fundamental to health care. In addition, nurse scholars Doane and Varcoe (2005, 2015) conceptualize relational nursing through a hermeneutic phenomenological lens recognizing that “relationships are not formed, they are lived” and that there is a “relational flow happening in every human encounter” (p. 179). Doane and Varcoe (2015) also contend that “people can only be understood in their relationship to their worlds, for it is only within their context that what people value and find significant is visible (p. 44).

Relational caring, in the way we have conceptualized it (Mitchell et al., 2020, 2021), is consistent with the aforementioned theories, as well as relational theory (Jordan, 2008, 2017; Jordan et al., 2004) where compassionate relationships are at the core of human wellness; human beings learn, evolve, and thrive best in mutual and reciprocal relationships (Dupuis, Gray, et al., 2016). We intentionally use relational *caring*, the verb, rather than relational *care* as a noun, to reflect a mutual process of caring rather than the dominant unilinear one where persons living with dementia are treated as passive recipients of care. We also recognize that relationships go beyond person-to-person relationships and exist within complex, dynamic, nested webs of connections among individuals and broader social, cultural, political, and environmental forces (e.g., stigma, policies) at multiple levels (e.g., family, organization, community, and culture), and that these forces play a critical role in supporting or denying the citizenship of people living with dementia (Dupuis, Gray, et al., 2016, Dupuis, Kontos, et al., 2016; Kontos et al., 2016, Kontos, Miller, & Kontos, 2017). Our conceptualization of relational caring philosophy brings together three major concepts: relationality (Dupuis, Gray, et al., 2016, Dupuis et al., 2018; Jordan et al., 2004; Kontos et al., 2016, Kontos, Miller, & Kontos, 2017); knowing otherwise (Olthius, 1997); and embodied selfhood (Kontos, 2012a, 2012b). Relational caring practice involves: being aware of and attentive to oneself, others, and relationships; showing empathy and curiosity by being responsive and emotionally present, moved by, and interested in others; being open to the role we play in relationships and alert to the potential consequences of our feelings and actions with others; being open to mutual influence and discovery and that learning and change flows both ways; being real, honest, and open with others in a respectful and compassionate way; being open to vulnerability and experiencing vulnerability as a place of potential growth rather than danger; sharing power and working in authentic partnership with others; respecting the abilities of all persons to live and have meaningful relationships; and tackling injustice and inequality not only by addressing barriers (i.e., implementing anti-oppressive policies and practices) but also by providing opportunities for self-expression, compassionate relationships, and flourishing (Dupuis, Gray, et al., 2016, p. 1; Kontos & Grigorovich, 2018).

In addition to the call for a new care ethic, there has been the growing awareness of the power of the arts to support verbal and non-verbal creative self-expression and connection, affect, and the opportunity to participate in activities that are meaningful (Dupuis, Kontos, et al., 2016; Kontos & Grigorovich, 2018; Swinnen & deMedeiros, 2018). Several authors direct attention to the possibilities of the arts to break the stronghold of the medicalized approach to dementia care (Basting, 2018; Bellass et al., 2019; Burnside et al., 2017; Camic et al., 2018; Melhuish et al., 2017; Sauer et al., 2016; Swinnen & de Medeiros, 2018; Whitehouse et al., 2018) and using the arts as a way to address stigma and expose inhumane policies and practices that cause unnecessary suffering for people living with dementia and their care partners (Dupuis et al., 2011, 2015; Dupuis, Kontos, et al., 2016; Jonas-Simpson et al., 2012; Kontos et al., 2018; Mitchell, Dupuis, et al., 2011; Mitchell, Jonas-Simpson, et al., 2011). Despite these benefits, the arts for the most part are restricted to an instrumental application—as a therapeutic intervention to reduce neuropsychiatric symptoms associated with dementia and improve cognitive and physical health outcomes (For a critique see: Basting, 2009; deMedeiros & Basting, 2014; Dupuis et al., 2012; Kontos & Grigorovich, 2018; Kontos et al., 2020). There have been important challenges to this instrumental reductionism with a call to support and nurture creativity in persons living with dementia in everyday life (Bellass et al., 2019; Kontos & Grigorovich, 2018; Kontos et al., 2020). Building on this broader approach to creativity, some scholars have advanced the notion of co-creativity to signal its relational and embodied nature (Bellass et al., 2019; Zeilig et al., 2018). However, as Kontos et al. (2020) have argued, “scholars who are advocating an embodied and relational approach to creativity are not fully engaging with this theoretical and empirical scholarship” (p. 3). Further, research to date that has explored the *experiences of relational engagement* with the arts has focused primarily on persons living with dementia leaving unexplored the relational experiences of others involved (e.g., family carers and staff).

## The study

### Aim

Our research aimed to address the gaps in the literature by focusing on the experiences of relational caring at an arts-based academy, guided by an embodied and relational caring ethic.

### Setting

The Academy operated Monday to Friday and was created for persons living with dementia seeking quality relationships, opportunities for creative self-expression, and learning through the arts, including music, theater, painting, poetry, and movement. The relational caring philosophy informed the design of all aspects of the Academy, including the physical and social space, all engagements, team and student education, as well as operations (Mitchell et al., 2020, 2021). For example, the physical space was intentionally designed to be home-like with a central, family-style dining area, and support free movement between different areas in the space including the “ballroom” for dance and movement, the “art cave” for other artistic engagements, the quiet room for reprieve, and an office; it was bright and warm with natural colors and natural light flowing through the windows that overlooked trees and a ravine.

Academy members were referred by their doctors, specialists, or memory clinics and could also self-refer. All persons living with dementia who were at different places on the dementia journey were welcome to attend. We refrained from labeling and assessing people, met them where they are at on the journey, and remained open to what is possible moment-to-moment. Academy members would attend once a week, two to three times a week, or every day, and some would attend in the morning only or the afternoon only, while others would stay the entire day from 9 a.m. to 4 p.m. A daily fee was required to attend the Academy, and the fee was subsidized by donations and a scholarship fund to support members who could not pay. On average, approximately 20 Academy members would attend a day with five staff, three artists, two to three family members, and up to eight personal support workers (PSWs). All were welcome in the space and encouraged to participate. Academy members were primarily from Judeo-Christian European backgrounds, while some were from other backgrounds such as, Asian, Caribbean, and African. Team members, leaders, students, artists, PSWs, and volunteers came from diverse backgrounds, including different countries of origin such as the United States, Nigeria, Philippines, South Africa, Russia, and England as examples.

All team members (i.e., program assistants, the coordinator, RN Health Coach, manager, and artists) were hired based on their fit with the relational caring philosophy. Ongoing workshops, mentorship, and in-the-moment discussions about the philosophy were provided to Academy team members and students by the Director and RN Health Coach. The arts were also used as a way to explore aspects of relational caring. For example, each month a core idea from one of our research studies was used to guide the theme of the month. Artists could ask, for example, what it is like to experience “unexpected friendliness,” and a poem, painting, or song may be created in the moment based on Academy members’ input. The philosophy was fundamental to every aspect of the Academy, *focusing all activities on relationships, meaningful engagements, and life enrichment.*

Academy arts-based engagements were structured so that members could choose to stay in one room or move to another. A typical day would begin with coffee and conversation with newspaper, crosswords, word puzzles, and art led by two staff. From table conversation and engagements, there was a shift to the “ballroom” for dance, a sing-along, and/or movement in one’s chair or standing. At the same time in the “art cave,” a ukulele lesson could be in full swing, or a water color lesson, or mural making. Again, the focus was not on the art itself but rather on relationships, meaningful engagements, and life enrichment.

Following the morning activities and classes, everyone came together for a hot lunch, where artists, students, the Director, and/or volunteers would engage in relationship and conversation with the Academy members. In the afternoon, there could be a music appreciation class with music history, a voice lesson and sing-along, a theater improv or theater devising class, or an art class. There is structure to the day and yet freedom within that structure was supported to go with the flow of Academy members, as inspired by complexity pedagogy (http://www.liberatingstructures.com/).

Given the Academy is funded through membership fees and primarily donations, there was a freedom from many of the typical restrictions of other dementia care settings, such as day programs. For instance, the Academy space was free of the task-oriented medical care assessments and practices that typically define other day programs and medical settings. For more details about the Academy, please see (Mitchell et al., 2020, 2021).

### Design

Given that we wanted to understand *the experience* of relational caring, van Manen’s (2014) qualitative phenomenological design was used to inform the study and address our two research objectives: (1) to explore how relationships are experienced when relational caring philosophy underpins practice, including arts-based engagements and (2) to understand the meaning of relationships that bring quality to day-to-day living.

### Participants

Following approval from Research Ethics Boards (the affiliated healthcare organization and university), researchers collaborated with the staff and coordinator at the Academy to recruit participants. An invitation to participate in the study was sent out to everyone affiliated with the Academy. To participate in this study, people needed to regularly attend the Academy and understand and speak English. A total of 25 participants volunteered to participate. Participants were predominately women (18). There were five persons living with dementia (two women and three men), eight family members (six women and two men), four staff (three women and one man), five artists (all women), one PSW (women), and two volunteers (one woman and one man). The participants living with dementia were at different places on the dementia journey and attended 2–3 times per week. The artist participants were professional artists from the community with media expertise in the fine artists, theater, movement, and music. The staff included program assistants, a coordinator, and a program leader. Five of the eight family members were related to the five participants living with dementia. The two volunteers attended the Academy 1–2 days per week, while the personal support worker attended with an Academy member several days per week.

### Data collection

As per our ethics protocol, all participants provided consent to participate prior to data collection. Persons living with dementia with the capacity to provide written consent did so, and consent by proxy was otherwise provided by carers. In addition to proxy consent, assent was obtained verbally by persons living with dementia prior to each interview. Capacity was determined in conversation with carers and team members.

The data were collected when the Academy entered the third year of operation. In-depth face-to-face interviews were conducted individually and in three small groups of participants who chose to be interviewed together (three artists, one couple, and two staff). Data were collected using an interview guide with open-ended questions, such as, “What is it like to be in relationship here at the Academy?”; “What has it been like for you here at the Academy?”; and an example question for team members, “What does relational caring mean to you?” Researchers sought further depth and clarity by asking participants at times to simply “go on” or “can you tell me more about that?” Interviews were video- and/or audio-taped.

#### Data analysis

The data analysis was informed by van Manen’s (2014) phenomenological thematic analysis process, which involved “recovering structures of meanings that are embodied and dramatized in human experience” (p. 319) and represented in the data. van Manen further describes the thematic analysis of lived experience as “a complex and creative process of insightful invention, discovery, and disclosure” (p. 320). With this approach in mind, we remained open to attend to the emergent meanings and insights in the data about the lived experience. Informed by van Manen’s “detailed reading approach” (p. 320), we worked individually with the sentences and words of the transcribed interviews to first identify the essences of meaning. Working collaboratively, these essences of meaning were then intertwined as thematic threads, and then woven into three thematic patterns that reflect the essence of lived experiences of relational caring at the Academy. Throughout this process, we used video segments to detect nuances of expression, meaning, and relations (Jonas-Simpson et al., 2015; Kontos, Miller, et al., 2017).

### Expressions of rigor

de Witt and Ploeg’s (2006) framework, which includes five expressions of rigor (i.e., balanced integration, openness, concreteness, resonance, and actualization), was used to ensure the rigor of our processes. Balanced integration was reflected in this study as philosophical concepts were evident and congruent throughout the study, from our choice of method, to the discussion. We also remained cognizant of the importance of integrating multiple voices in our representations of the data. We openly presented our systematic approach and multiple decisions throughout the process (e.g., recruitment, interview questions, and data analysis process). Our study findings present new ideas to guide practice that are both relational and arts-based, which reflects both rigor and concreteness. Resonance was confirmed by external readers steeped in relational arts-based practices. Finally, actualization reflects the “future realization of the study findings” (p. 215) and this we believe will emerge as people consider our findings for practice.

## Findings

Our findings, expressed as thematic patterns, reflect how relationships are experienced when relational caring underpins practice, including arts-based practices, and help us to understand the meaning of relationships that bring quality to day-to-day living. These thematic patterns are not distinct but rather are interwoven patterns emerging from several thematic threads that create an overall description or tapestry of what it is like to experience a relational ethic of care at an arts-based academy for persons living with dementia.

### Freedom and fluid engagement inspire a connected spontaneous liveliness

The thematic pattern *freedom and fluid engagement inspire a connected spontaneous liveliness* is metaphorically reflected in the phenomenon of murmurations, the movement birds engage in when hundreds or thousands fly and land in rhythm as though one entity. Similarly, the way people move together in the Academy space is in tune with everyone’s way of being that continually shifts and changes. The freedom and fluid engagement are possible with the physical and philosophical design of the Academy, a flexible space, where movement emerges with the interests of the people who are free to join in, in the way they wish. It is always fluid, going in one direction and then another. As a volunteer said, “it is about life being, you know, lived…It’s the flow, the freedom of it going wherever it does.” A participant living with dementia highlighted the freedom of choice:

Just being here is enough… I do arts or …sit in on dancing and I’m allowed to dance if I want or not to dance if I want. It’s a good feeling being here... It means additional life.

 The freedom of movement inspires the emergent liveliness, joy, and enrichment. The structure of the day enabled the freedom for Academy members to move from one kind of engagement to another. For instance, art at one table might spawn a song or dance that connects with a cross word puzzle or picture. If an Academy member did not wish to join group engagements, they could instead, for example, join a team member, volunteer or student playing ukulele, painting, or constructing innovative art pieces out of wood or yarn in the art cave. The relational space itself created this sense of freedom and fluidity. In-the-moment creative play is emergent, unexpected, spontaneous, and organic. One artist said, “when you’re in flow…it’s just creating itself with [members’] input, with my input…it just built on itself …we moved that into our bodies… improvisational movement, I mean, just all different things just flowed out of it, and by the end of class we were like, ‘wow, we’re all more connected than we’ve ever been in this class before,’ and I felt joyful.” A volunteer said:

…it’s very easygoing so once you hop on this flow and you go with it you know, it takes you some places…so I guess it’s the element of surprise, I really like it here…it’s the flow, the freedom of it going wherever it does…we do not differ between people with dementia or without you know, we just go with it…people do what they want…they feel free and safe… and if you don’t want to participate we can do something else; it is incredible!

 Another artist said, “different energies will pop up…sometimes I’m feeding off the energy… it may go a different direction than I had originally planned because we’re being in the moment and spontaneous and…so I appreciate that there’s lots of laughter and there’s lots of opportunity to be creative… not stick to a script.” One participant living with dementia said, “…the way we were dancing, the way we were moving and going like this, up here, going up here on our head. And, everybody who was there, stopped and they were at their tables. And their looking at the two of us, we’re dancing here, we’re dancing there.” Enjoying one another becomes possible when each member of the Academy is accepted for who they are while remaining open to new discoveries.

### Embracing difference invites discovery with generous inclusivity

The thematic pattern *embracing difference invites discovery with generous inclusivity* captures the openness that encircles everyone in the space and that also includes the learning, discovery, and mutuality that emerges when difference and vulnerability are viewed as opportunity. One participant living with dementia expressed this inclusivity in the way he felt welcomed at the Academy, “The people in here, really, are all very nice. They will [say] ‘Hello!’ and they wear their hands like that [raises his hand to gesture hello] and they take their caps off like that. It is extremely nice.” This thematic pattern also captures how *all* were welcomed in the space. One family member described the immediate embrace she experienced:

I came in and immediately I knew because I was greeted so warmly…almost lovingly, and yet we didn’t know each other but it filled me with such confidence for [names spouse]. I felt like it was inclusive; it was [names spouse] and I coming, it wasn’t just a drop off, and that made such a difference to me.

 Another family member compared her experiences at the Academy with other day programs and described it as: “a feeling of embrace when you walk in as opposed to a feeling of a wall of how do I, you know, get through this wall?” A volunteer also described how difference without judgment is embraced and how everyone is included in generous open ways and is viewed as having something to contribute:

[B]eing like a family we do have respect for each other… There is no like distinguishing between who does and who doesn’t have memory loss, we all have moments you know, but the thing is that with the memory loss, without memory loss we are just bundles of something that you know, was meant to be, like personality-wise we’re interesting…

 Embracing difference opened opportunities for discovery. A participant living with dementia stated, “more people are here, that makes a difference. People are not all the same…there is a novelty of not knowing what people are going to say, so it’s great, it really works well, you never know who you’re going to see and what you’re going to be doing.” One staff member said, “It’s been a lot of discovery... I’m learning just as they are learning…not just learning to get to know the individual, I’m learning a lot about myself.” Another staff member said:

I would say the diversity sort of drives…the atmosphere, because I can’t imagine a situation where we all like the same thing, feel the same way, experience things the same… but when you have…different views, different reactions, different thoughts, it makes everything more lively…

 Emerging from the unconditional embrace of difference is a mutual affection and sense of trust where people feel free to express themselves authentically.

### Mutual affection brings forth trust and genuine expression

Participants, regardless of who they were all described feeling authentically accepted, free to express themselves, to give and receive. Many described feeling love and that the Academy was another family where unexpected kindnesses would emerge with a feeling of comfort. One participant living with dementia shared, “you just feel good!;” while an artist said, “it hums in you.”

The experience of a warm welcome, feeling included, when arriving at the Academy co-creates a sense of trust, safety, and relief that is often felt immediately and enhanced over time as relationships grow. One family member said, “they know our names, which is lovely, makes us feel comfortable very quickly” while another family member said, “there’s a great feeling of comfort for me, knowing that when he comes here, he feels comfortable.” One family member said she was surprised “how quickly” her husband became comfortable while another family member said, “What is the feeling? I think it is warmth, I think its acceptance, and I think that they really put people at ease.”

The foundation of love, loving and being loved, was described by a participant living with dementia who said, “I feel a friendliness that makes me feel wonderful…I feel such a nice warmth that I look forward to coming here… I feel love…I get hugs, I hug back…I feel like you’re happy to have me here and I’m happy to be here because you love me, I love you back.” A personal support worker similarly shared this sentiment: “being there with these people at the [Academy], the warmth, the love, the care, the concern, it makes me very happy that I’m in this environment and I’m able to share my love also.”

The trust emerging from the mutual affection also supports the freedom of genuine expression. One family member said, “I have a great deal of trust…they make him feel important, that there’s nothing he can’t do.” Another family member echoed this feeling of support as she described how her mother, an Academy member and poet, was acknowledged for the unique person she is: “She feels very acknowledged here…for …who she is. They think she’s very creative and you know, vibrant, and I think, that’s showing more as she’s been coming more.” This feeling of trust that supports creative expression was felt by all at the Academy. One artist said, “It’s just a great spirit of creative discovery…I feel like I can bring who I am, you know, I feel like the participants feel that too, they can be who they are, have some fun and maybe some courage to step outside the box, try something they might not necessarily do but feel safe, you know there is no judgment.” One staff member said, “when [members] try out the new thing, then they discover that they’re able to enjoy it, you know, give it a try…just that encouragement from the rest of the participants; …it gives them that confidence to say, ‘oh yeah, I can do this and this looks good!” One participant living with dementia said, “being at a place where you can actually do things good and there not when you sort of get and say ‘oh I can’t do that…’ Yeah when I am here I just feel like, I’m not trying to be a showoff—but you just feel good.”

## Discussion

Findings from our study capture experiences of relational caring and align with the literature that describes what is possible when a relational philosophy and ethic of care guides practice (Armstrong & Lowndes, 2018; Dupuis, Kontos, et al., 2016, Dupuis et al., 2019; Greenwood & Archdall, 2014; Kontos, Miller, et al., 2017; Mitchell et al., 2020, 2021). Also, our findings position the arts as meaningful mediums for relational caring (Basting, 2018; Bellass et al., 2019; Kontos et al., 2020), which support human flourishing (Kontos & Grigorovich, 2018; Whitehouse et al., 2018). Methodologically, our study also addresses a gap in the arts-based literature by providing research about a *collective* experience of engaging in the arts that is grounded in a relational caring ethic.

Our finding, *freedom and fluid engagement inspire a connected spontaneous liveliness,* highlights how experiences of relational caring are supported and enhanced when persons living with dementia have the freedom to choose how they engage at an arts-based academy, with a home-like environment. This finding is consistent with some of the “promising practices” identified by Armstrong and Lowndes (2018) such as supporting “residents’ freedom of movement and their ability to socialize and engage in meaningful activities as they would if they were at home” (Lowndes and Struthers, p. 73). An important difference with our findings is that the Academy’s foundation is an arts-based relational model of caring, which goes beyond a social model of care (Mitchell, Dupuis, et al., 2020; Mitchell, Jonas-Simpson, et al., 2020). The freedom and fluidity identified in this theme reflects Daly’s (2018) call for activities to meet people “where they are at—in pace, structure, timing, and content” (p. 63). At the Academy, a member can spontaneously engage in the arts from wherever they are in the moment and in whatever way they choose while also physically moving from one engagement to another, such as from musical engagement to theatrical engagement.

In our study, the freedom and fluidity of the Academy space also inspired a *connected spontaneous liveliness*. This vibrant spontaneity is an important dimension of relational caring that was not evident in the other evaluations of relational practices (e.g., Brown Wilson et al., 2013; Dewar & Nolan, 2013), perhaps due to the structural and organizational constraints of the settings. However, Daly (2018) reported a home in Germany “where spontaneous music was a part of the day and where music, dancing, and rum punch were also part of the fun” (p. 62) Learning to let go of control and embrace spontaneity to heighten engagement was also discovered as significant in arts-based programs such as the Penelope project (Basting et al., 2016). Letting go of control highlights how the arts enhance relational practices, which support the spontaneous liveliness not only of art practitioners, but of all in practice settings, including staff, family members, volunteers, and others. Whitehouse et al. (2018) call for funding to support building capacity among all practitioners regarding the arts; however, we oppose the naming of this developed skill as “social prescribing,” which perpetuates the medical model and aligns with an individualistic model of care where experts prescribe what arts-based activity or engagement a person living with dementia needs. Even the term “activities” has been replaced with “engagements” at the Academy to ensure the structure remains relational, open, engaging, and emergent, which *invites connected spontaneous aliveness*. This approach aligns with one of Basting’s (2018) core elements of a creative community of care, that is, *immersion*, whereby the arts are not simply designated to certain activity times but rather are “infused into daily relationships and the very air of the place” (p. 750).

Basting (2018) raises an important point about how “infusing creativity into the very fabric of the community itself enabled people to see themselves and each other differently—as having potential for meaningfulness” (p. 751). This idea links to our theme, *embracing difference invites discovery with generous inclusivity*. In addition, embracing difference was identified in our findings as essential for discovery when people experienced relational caring as acceptance for who they are, which created a feeling of trust and courage to try new things and express themselves in new ways. Dupuis, Kontos et al. (2016) also described how relational arts can prompt new discovery and transformation that can challenge stigma of dementia and inspire actions to promote social justice. However, Basting (2018) notes that most arts-based projects are a one-time event or short term, which can impede opportunities for growth and learning. At the Academy, by contrast, the arts are always present and embedded mediums for relational caring precisely because of how fundamental they are to embracing difference and supporting inclusion, enhancing relationships and relationality, facilitating self-expression and discovery, and enhancing the quality of day-to-day living.

Relational caring was experienced as mutual affection, which brought forth a sense of safety and *trust and genuine expression*. The *mutual affection* shown at the Academy refers to the ways that members expressed caring for others and to the intimacy and love felt; indeed, the Academy was often referred to by members as a second family. Feeling safe while trusting others freed participants to genuinely express themselves. Based on our findings, close, trusting, compassionate relationships were essential to the experience of relational caring, which, in turn, is essential for thriving. Further, these relationships were supported by the spontaneous and fluid nature of engagement with the arts. Participants in our study described how relationships are created and nurtured when singing, dancing, painting, or engaging in theatrical improv together. This finding suggests that the arts could be incorporated into the day-to-day of long-term care (LTC) homes to support relationships and life enrichment just as how Lowndes and Struthers (2018) described how staff in Germany supported relationships with residents in LTC homes through singing, conversing, playing games, and making crafts.

There are countries leading transformation in dementia care by establishing policies, protocols, and promising practices that push beyond the biomedical domination (Armstrong & Lowndes, 2018; Camic et al., 2018; Greenwood & Archdall, 2014; Whitehouse et al., 2018). Power relations and issues of evaluation also differ in the literature and the differences prompt us to ask more critical questions about the value and purpose of the arts. We consider a question raised by Bellass et al. (2019) about the context of arts-based practices. Namely, can arts-based practices thrive in a clinical context where power relations are well established in ways that restrain possibilities in dementia caring? We argue, based on our findings, that people thrive *when arts-based practices are grounded in relational theory and when relational theory is lived through arts-based mediums*. Without the theoretical grounding of a relational ethic of care, arts-based practices can become yet another expressed power relation. We see this in most dementia care contexts where art is only valued as an intervention (Dupuis, Whyte, et al., 2012). So, it is the joining of both a relational caring philosophy with arts-based modalities as mediums for relational caring experiences that we found made a difference to relationships and overall life quality among all persons affiliated with the Academy.

Methodologically, our study fills some of the arts-based and relational caring research gaps. Bellass et al. (2019) concludes that the existing body of research “tells us more about the experience of creative interventions than it does the experience of being creative in daily life with dementia…it tells us very little about the individual’s experience of creativity” (p. 2803). Our findings share the experiences of relational caring and engaging in creativity day-to-day with others in a way that supports difference with radical inclusivity. While most research focuses on both relational care and arts-based activities as interventions, ours focuses on process and quality of experiences of engagement. Further, our research examined experiences of all those affiliated with the Academy and highlights the possibilities of the arts for enhancing relationships and community-building among diverse individuals. Also, while all participants living with dementia were white, we included both men and women at different places on the dementia journey, whereas most studies are based on white women and persons living with mild dementia (Bellass et al., 2019). Consistent with Nolan et al. (2002, 2004, 2006), our research further demonstrates the importance of attending to the wellness of everyone in dementia settings and the potential of arts-based practices grounded in relational caring to support the flourishing of all in these settings.

The uniqueness of the Academy raises issues of transferability. The Academy, funded by donors and members, is not constrained by the regulatory structure of LTC homes and day programs that often create tensions between a relational caring philosophy and practice (Dupuis et al., 2019). While there are many freedoms that come with this funding structure, there are also tensions, similar and yet different from “equity in access” (Armstrong, 2018, p. 29). The Academy makes efforts to ameliorate these tensions by offering a scholarship fund for those who require support to attend. Another reason transferability may be an issue is that the Academy’s organizational and physical structures support relational practices. There is no “strict division of labor” (Armstrong, p. 29) and the space is free of the task-oriented medical care practices that typically define other community settings and LTC homes. Our findings may, however, be transferrable to LTC settings, both residential and day programs, that embrace a relational caring ethic and what Lowndes and Struthers (2018) identified: a team-based approach where the division of labor is blurred and where relational caring needs are prioritized over biomedical assessments and functional care; and where there is freedom to move and socialize for all (p. 72). As new settings and programs are developed, grounding them in a relational caring philosophy from the beginning and providing ongoing support of the principles will better support the transfer of the philosophy into practice. For example, relational principles have been translated to a virtual platform (thebitovemethod.com), and we are currently conducting research to explore relational caring experiences in those virtual arts spaces.

## Conclusion

Our research captures experiences of relational caring and shows what is possible when an arts-based relational ethic of caring is embraced by all those affiliated with an Academy for persons living with dementia. We agree with Basting (2018) that the “arts have and are continuing to emerge as a powerful tool to foster [the] transformational efforts” of the age- and dementia-friendly movements (p. 745). We hope our research contributes to significant culture change in community-based and LTC homes where the arts as a medium for relational caring practices can support everyone to flourish.
